# Detection and Analysis of Antidiarrheal Genes and Immune Factors in Various Shanghai Pig Breeds

**DOI:** 10.3390/biom14050595

**Published:** 2024-05-17

**Authors:** Jinyong Zhou, Fuqin Liu, Mengqian He, Jun Gao, Caifeng Wu, Yeqing Gan, Yi Bian, Jinliang Wei, Weijian Zhang, Wengang Zhang, Xuejun Han, Jianjun Dai, Lingwei Sun

**Affiliations:** 1 Shanghai Municipal Key Laboratory of Agri-Genetics and Breeding, Institute of Animal Husbandry and Veterinary Science, Shanghai Academy of Agricultural Sciences, Shanghai 201106, China; jinyzhou@126.com (J.Z.); liufuqin0518@163.com (F.L.); he1037247863@163.com (M.H.); gaojun@saas.sh.cn (J.G.); wucaifengwcf@163.com (C.W.); 2 Key Laboratory of Livestock and Poultry Resources (Pig) Evaluation and Utilization, Ministry of Agriculture and Rural Affairs, Shanghai 201106, China; 3 Shanghai Jiading Municipal Centre for Disease Control and Prevention, Shanghai 201899, China; 18930381338@163.com (Y.G.); kytdyx2022@163.com (Y.B.); 13671798163@163.com (J.W.); 4 Shanghai Municipal Centre for Disease Control and Prevention, Shanghai 200051, China; 18275144179@163.com (W.Z.); verkil8586@163.com (W.Z.); 5 Shanghai Engineering Research Center of Breeding Pig, Shanghai 201106, China; 13887223118@163.com

**Keywords:** Shanghai white pig, Fengjing pig, Shawutou pig, Meishan pig, Pudong white pig, gene, immunity factors

## Abstract

The aim of this study was to identify effective genetic markers for the Antigen Processing Associated Transporter 1 (*TAP1*), α (1,2) Fucosyltransferase 1 (*FUT1*), Natural Resistance Associated Macrophage Protein 1 (*NRAMP1*), Mucin 4 (*MUC4*) and Mucin 13 (*MUC13*) diarrhea-resistance genes in the local pig breeds, namely Shanghai white pigs, Fengjing pigs, Shawutou pigs, Meishan pigs and Pudong white pigs, to provide a reference for the characterization of local pig breed resources in Shanghai. Polymerase chain reaction–restriction fragment length polymorphism (PCR-RFLR) and sequence sequencing were applied to analyze the polymorphisms of the above genes and to explore the effects on the immunity of Shanghai local pig breeds in conjunction with some immunity factors. The results showed that both *TAP1* and *MUC4* genes had antidiarrheal genotype GG in the five pig breeds, AG and GG genotypes of the *FUT1* gene were detected in Pudong white pigs, AA antidiarrheal genes of the *NRAMP1* gene were detected in Meishan pigs, the AB type of the *NRAMP1* gene was detected in Pudong white pigs, and antidiarrheal genotype GG of the *MUC13* gene was only detected in Shanghai white pigs. The *MUC13* antidiarrhea genotype GG was only detected in Shanghai white pigs. The *TAP1* gene was moderately polymorphic in Shanghai white pigs, Fengjing pigs, Shawutou pigs, Meishan pigs and Pudong white pigs, among which *TAP1* in Shanghai white pigs and Shawutou pigs did not satisfy the Hardy–Weinberg equilibrium. The *FUT1* gene of Pudong white pigs was in a state of low polymorphism. *NRAMP1* of Meishan pigs and Pudong white pigs was in a state of moderate polymorphism, which did not satisfy the Hardy–Weinberg equilibrium. The *MUC4* genes of Shanghai white pigs and Pudong white pigs were in a state of low polymorphism, and the *MUC4* genes of Fengjing pigs and Shawutou pigs were in a state of moderate polymorphism, and the *MUC4* genes of Fengjing pigs and Pudong white pigs did not satisfy the Hardy–Weinberg equilibrium. The *MUC13* gene of Shanghai white pigs and Pudong white pigs was in a state of moderate polymorphism. Meishan pigs had higher levels of IL-2, IL-10, IgG and TNF-α, and Pudong white pigs had higher levels of IL-12 than the other pigs. The level of interleukin 12 (IL-12) was significantly higher in the AA genotype of the *MUC13* gene of Shanghai white pigs than in the AG genotype. The indicator of tumor necrosis factor alpha (TNF-α) in the AA genotype of the *TAP1* gene of Fengjing pigs was significantly higher than that of the GG and AG genotypes. The indicator of IL-12 in the AG genotype of the Shawutou pig *TAP1* gene was significantly higher than that of the GG genotype. The level of TNF-α in the AA genotype of the *NRAMP1* gene of Meishan pigs was markedly higher than that of the AB genotype. The IL-2 level of the AG type of the *FUT1* gene was obviously higher than that of the GG type of Pudong white pigs, the IL-2 level of the AA type of the *MUC4* gene was dramatically higher than that of the AG type, and the IgG level of the GG type of the *MUC13* gene was apparently higher than that of the AG type. The results of this study are of great significance in guiding the antidiarrhea breeding and molecular selection of Shanghai white pigs, Fengjing pigs, Shawutou pigs, Meishan pigs and Pudong white pigs and laying the foundation for future antidiarrhea breeding of various local pig breeds in Shanghai.

## 1. Introduction

China, as a large pig-raising country, has a fairly high stock of pigs, and swine diarrheal disease is frequent throughout the year. The high incidence of this disease, which causes serious economic losses, is a major problem that seriously restricts the development of the pig farming industry in China and even around the world [1]. The ultra-high feed-to-meat ratio, growth retardation, delayed farrowing time and developmental stagnation (stiff pigs) caused by diarrhea are also the key to the economic income of pig farms. Although there are vaccinations, medications, microecological agents and antimicrobial peptides for piglet diarrhea, they have not solved piglet diarrhea fundamentally. Therefore, the application of disease-resistant breeding to screen disease-resistant strains and enhance a pig’s own ability to resist diarrhea can fundamentally solve the economic losses caused by pig diarrheal diseases. Currently, the detection and analysis of allele frequencies of antidiarrhea-related genes in pigs through molecular breeding technology, which can be used to guide pig breeding, can improve the overall antidiarrhea ability of pigs from the perspective of breeding.

It is known that the α (1,2) fucosyltransferase gene 1 (*FUT1*) and antigen processing-associated transporter gene 1 (*TAP1*) are the main candidate genes for enterotoxigenic Escherichia coli (ETEC) F18 [2]. Meijerink et al. found that the *FUT1* gene had a G → A base mutation at the 307 bp site and that the AA genotype of Swiss long white pigs was resistant to ETEC F18 infection [3]. Luc D D et al. reported that *FUT1* was a candidate gene controlling the adhesion of *E. coli* F18 receptor and that AA genotypes of pigs were resistant to enterotoxigenic *E. coli* F18 [4]. Kim et al. further demonstrated that individuals with the AA genotype of the *FUT1* gene had almost twice the survival rate of piglets than GG individuals [5]. Consequently, *FUT1* can be used as an effective gene for antidiarrheal breeding. Zhang et al. demonstrated that GG individuals with the *TAP1* gene were more resistant to F18 ETEC infection in pig breeds such as large white and Pudong white [6]. They showed that the *TAP1* gene can be used as one of the effective genetic markers for breeding pigs for diarrhea resistance.

The Natural Resistance Associated Macrophage Protein 1 (*NRAMP1*) gene is able to influence the disease resistance performance of animal organisms, and the base C → A at 111 bp on intron 6 of the porcine *NRAMP1* gene yielded three genotypes (AA, BB, AB), with the AA genotype being the dominant antidiarrhea gene [7]. Chen et al. investigated the relationship between polymorphisms of the *NRAMP1* gene of large white pigs and its immune function, indicating that *NRAMP1* is a good candidate gene for disease resistance breeding [8]. Studies have confirmed that the Mucin 4 *(MUC4)* and Mucin 13 (*MUC13*) genes are the most important candidate genes for the ETEC F4ab/F4ac receptor [9,10]. Rampoldi et al. reported that there is an interlocking disequilibrium between the porcine ETEC Fab/4ac receptor motifs and intron 7 of *MUC4* [11]. However, Peng et al. showed that susceptibility or resistance to ETEC F4ab/ac infection in pigs was obviously correlated with the A → G mutation at site G243 in intron 17 of the *MUC4* gene and, combined with prophylactic information, demonstrated that the GG type of the *MUC4* gene was an antidiarrhea genotype [12]. Zhang et al. found the presence of a G → A mutation in the rs319699771 locus of the *MUC13* gene, with a GG-type diarrhea-resistant genotype, and the ability to identify diarrhea-resistant individuals by identifying mutations at this locus [13]. In addition, the *MUC4* and the *MUC13* genes were considered as one part of the most promising candidates for the F4ab/F4ac receptor [14].

In this study, we screened the genetic polymorphisms of *TAP1*, *FUT1*, *NRAMP1*, *MUC4* and *MUC13* genes in Shanghai white pigs, Fengjing pigs, Shawutou pigs, Meishan pigs and Pudong white pigs. Testing results combined with some hematological immune factors assay results (immunoglobulin (IgG), interleukin 2 (IL-2), interleukin 6 (IL-6), interleukin 10 (IL-10), interleukin 12 (IL-12), tumor necrosis factor ɑ (TNF-ɑ), interferon γ (IFN-γ)) were selected and used to analyze the antidiarrheal capacity of the above pig breeds and to further analyze the population diversity, in order to lay the foundation for the improvement of antidiarrheal breeding of the above pig breeds.

## 2. Materials and Methods

### 2.1. Animals and Samples

A total of 150 reserve breeding gilts were selected for this study, including five local pig breeds in Shanghai, China: Shanghai white pigs (*n* = 30), Fengjing pigs (*n* = 30), Shawutou pigs (*n* = 30), Meishan pigs (*n* = 30) and Pudong white pigs (*n* = 30). All groups of pigs were of similar weight and age (Supplemental Table S1). These gilts were kept in individual pens, where they were fed daily according to the same standards, were given fresh drinking water freely, and underwent disease control according to the Chinese Guide to Pig Feeding Standards. For each pig, 10 mL blood samples were collected individually from the jugular vein and divided into two vials: 5 mL blood samples were taken in BD Vacutainer^®^ blood collection tubes without anticoagulants (Becton Dickinson, Heidelberg, Germany), centrifuged for 5 min at 12,000× *g* at room temperature, and stored at −20 °C until biochemical analysis; 5 mL blood samples were taken into BD Vacutainer^®^ blood collection tubes (EDTA spray-coated) and stored at −20 °C until for subsequent DNA extraction.

### 2.2. Determination of Blood Immune Factors

The absorbance of the samples was determined using ELISA immune factor assay kits (Elabscience Biotechnology Co., Ltd., Wuhan, China), and the absorbance values were used to draw standard curves for the measurement of the following immune factors: IgG (DG50132P-96T), IL-2 (DG50032P-96T), IL-6 (DG50031P-96T), IL-10 (DG50034P-96T), IL-12 (DG50055P-96T), TNF-α (DG50014P-96T) and INF-γ (DG50141P-96T). The measured indices were used as an evaluation of the immune response and general disease resistance of Shanghai white pigs, Fengjing pigs, Shawutou pigs, Meishan pigs and Pudong white pigs.

### 2.3. DNA Extraction and Concentration Determination

Genome DNA was extracted from blood samples using the TIANamp blood DNA kits (TIANGEN, Beijing, China). The DNA concentration was determined using an ultraviolet spectrophotometer (Eppendorf product), and the samples with a concentration range of 80–250 µg/µL were selected for subsequent experiments. The quality of extracted DNA was examined by 1.5% agarose gel electrophoresis. The DNA was uniformly diluted to 50 ng/μL and stored at −20 °C in a refrigerator.

### 2.4. Genotyping Analysis

Genotyping was performed using polymerase chain reaction–restriction fragment length polymorphism (PCR-RFLP). The primers for the *FUT1*, *NRAMP1*, *TAP1*, *MUC13* and *MUC4* genes were designed using Premier 6 software by searching the related sequences on NCBI, and the primers were synthesized by Shanghai Sangong Bioengineering Co. Ltd., Shanghai, China (the sequences of primers are listed in Supplemental Table S2).

The PCR reaction system of 20 μL contained template DNA 2 μL, 2xM5 Taq HiFi PCR Mix 10 μL, forward primer (10 μm) 0.5 μL, reverse primer (10 μm) 0.5 μL and nuclease-free ddH_2_O supplemented to 20 μL. The PCR reaction conditions were as follows: pre-denaturation at 94 °C for 4 min, followed by denaturation at 94 °C for 30 s, annealing for 30 s at 46 °C for *TAP1*, 55 °C for *FUT1*, 64 °C for *NRAMP1*, 50 °C for *MUC4*, and 60 °C for *MUC13*, and extension at 72 °C for 60 s for *TAP1* and *FUT1*, with a total of 30 cycles, for *NRAMP1*, with a total of 45 s, and a total of 29 cycles, *MUC4* for 15 s, a total of 34 cycles, *MUC13* for 45 s, a total of 35 cycles.

The PCR products of the *TAP1* gene were digested using the restriction enzymes *MboI* (New England BioLabs, Ipswich, MA, USA). The total system of the digestion reaction was 10 µL, including PCR amplification product 0.4 µL, restriction enzyme (10 U/µL) 0.4 µL, 10 × NE buffer 1 µL, ddH_2_O 8.2 µL. The reaction was kept at a constant temperature of 37 °C for 2 h. The digestion reaction was stopped by incubation at 65 °C for 20 min and was analyzed by 1.5% agarose gel electrophoresis.

The PCR products of the *FUT1* and *MUC4* genes were digested by *HhaI* (Boehringer Ingelheim, Mannheim, Germany). The total system of the digestion reaction was 10 µL, including PCR amplification product 1 µL (500 ng/µL), 10 × RE buffer 1 µL, acetylated BSA 0.1 µL (10 µg/µL), recommendation enzyme 0.1 µL and BSA (10 µg/µL) 0.1 µL. The system was set at 37 °C for 2 h; after the addition of 4 µL of 6 × loading buffer, the reaction was analyzed by agarose gel electrophoresis at 1.5%.

The PCR products of the *NRAMP1* gene were digested by *NdeI* (New England BioLabs, Ipswich, MA, USA). The total system of enzyme digestion was 10 µL, including 500 ng/µL of PCR amplification product 1 µL, 10 × RE buffer 1 µL, acetylated BSA (10 µg/µL) 0.1 µL, restriction enzyme 0.25 µL (10 U/µL) and ddH_2_O 7.65 µL, and then 4 µL (10 U/µL) of *NdeI* was added to the reaction for 2 h at 37 °C. After the addition of 4 µL of 6 × loading buffer, the reaction was analyzed by agarose gel electrophoresis at 1.5%.

The PCR products of *MUC13* had no specific endonuclease; no digestion was performed.

The PCR products were purified and sequenced by Shanghai DNA Biotechnologies company. The sequence results were analyzed using DNAstar software (version 11.0, DNAstar, Madison, WI, USA).

### 2.5. Statistical Analysis

The sequencing results were compared using BLAST in the NCBI system to identify SNP loci. The calculation of allele and genotype frequencies for each gene was analyzed by Hardy–Weinberg equilibrium and chi-square test of independence using the fitness test. Parameters related to genetic variation and differentiation within the population were obtained by Popgene software (version 1.31). The genotypic frequency and allele frequency of the population (Ho), heterozygosity (He), effective allele numbers (Ne) and polymorphism information content (PIC) were calculated using online software (http://www.msrcall.com/Gdicall.aspx, accessed on 10 January 2023).

## 3. Results

### 3.1. Genes’ PCR Amplification

The DNA extracted from Shanghai white pigs, Fengjing pigs, Shawutou pigs, Meishan pigs and Pudong white pigs was amplified according to the primers of the *TAP1*, *FUT1*, *NRAMP1*, *MUC4* and *MUC13* genes. As shown in Figure S1, the sizes of amplified DNA fragments were consistent with the design.

### 3.2. Results of Restriction Enzyme Digestion

**Figure 1:**
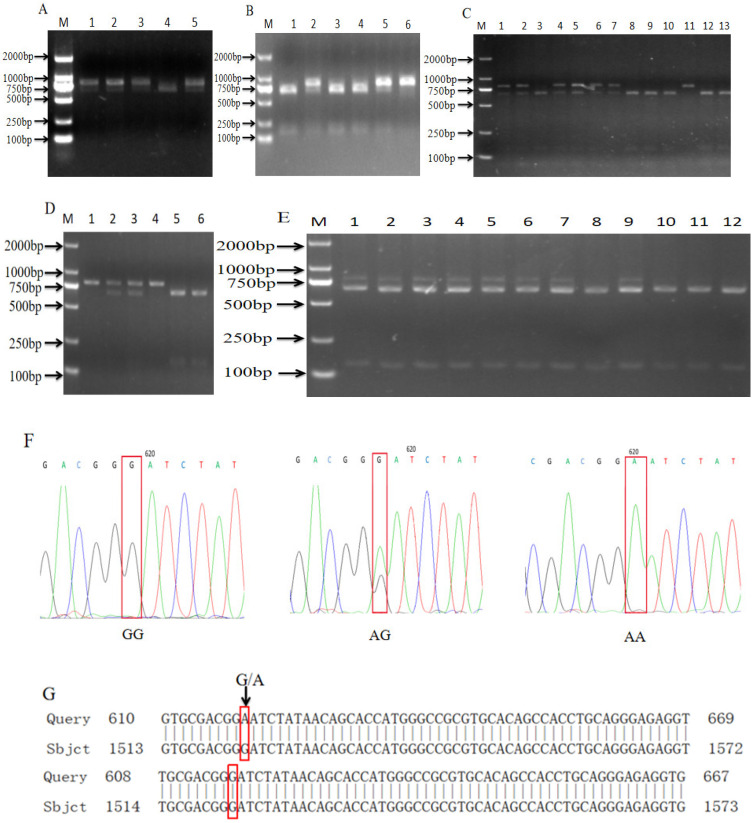
Enzymatic digestion and sequencing results of *TAP1* gene. (**A**–**E**) Enzymatic digestion results of Shanghai white pig (**A**), Fengjing pig (**B**), Shawutou pig (**C**), Meishan pig (**D**) and Pudong white pig (**E**). (**F**) Sequencing peak plot. (**G**) Comparison map of NCBI sequence of *TAP1* gene.

Figure 1A–E show the PCR-RFLP verification of the AG and GG genotypes of *TAP1* in Shanghai white pigs and Pudong white pigs and the AA, GG and AG genotypes of *TAP1* in Fengjing pigs and Shawutou pigs. According to the enzyme digestion results, the PCR products were sequenced and compared (Figure 1F). It was found that the sequence of GG-type individuals was consistent with the sequence of AK396698.1 in NCBI, which was defined as the wild type. Conversely, the GG type was found to have a single-base mutation of G → A at position 729, which was consistent with the enzymatic results (Figure 1G).

**Figure 2:**
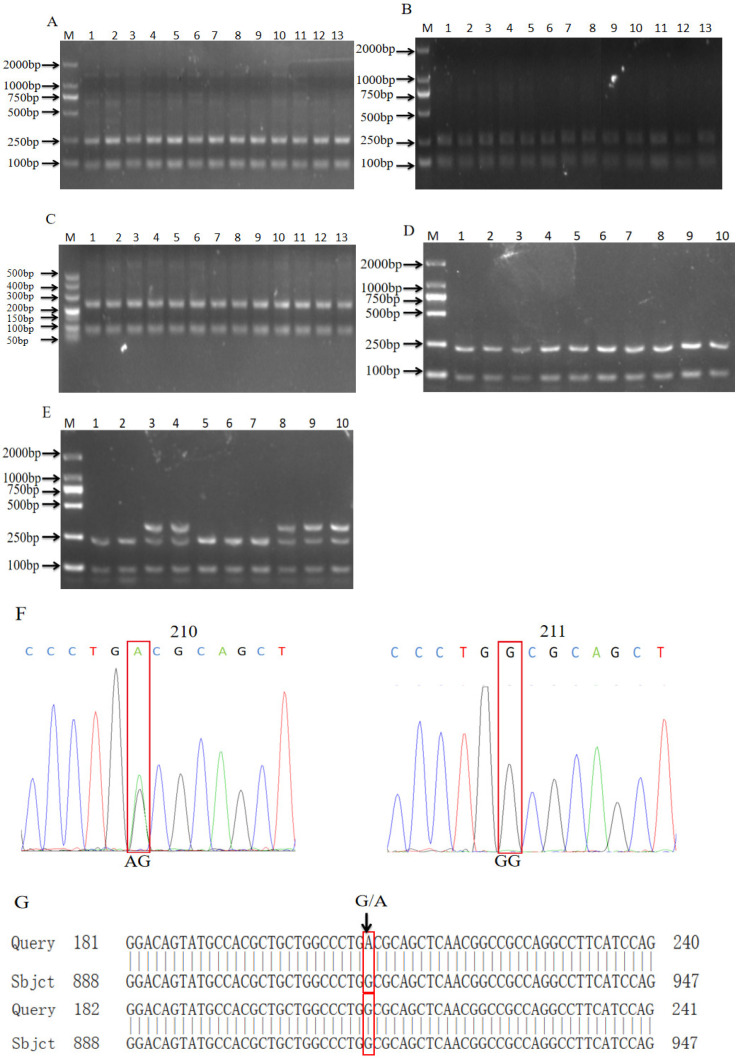
Enzymatic digestion and sequencing results of *FUT1* gene. (**A**–**E**) Enzymatic digestion results of Shanghai white pig (**A**), Fengjing pig (**B**), Shawutou pig (**C**), Meishan pig (**D**) and Pudong white pig (**E**). (**F**) Sequencing peak plot. (**G**) Comparison map of NCBI sequence of *FUT1* gene.

As shown in Figure 2A–E, we found that the *FUT1* genes of the Pudong white pigs had two genotypes (AG and GG), but the other pig breeds only had one type of band (GG). According to the enzyme digestion results, the PCR products were sequenced and compared (Figure 2F). The sequence of the GG-type individual was consistent with the sequence of U70883.2 in NCBI, which was consistent with the enzyme digestion results (Figure 2G).

**Figure 3:**
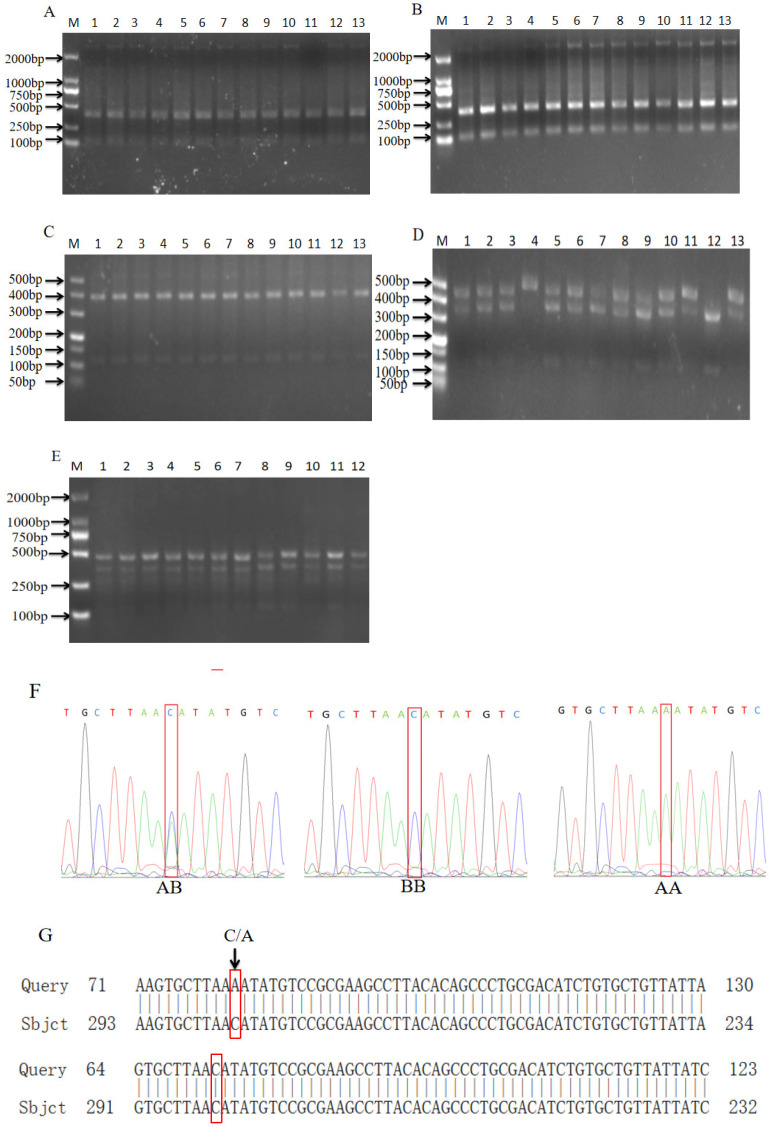
Enzymatic digestion and sequencing results of *NRAMP1* gene. (**A**–**E**) Enzymatic digestion results of Shanghai white pig (**A**), Fengjing pig (**B**), Shawutou pig (**C**), Meishan pig (**D**) and Pudong white pig (**E**). (**F**) Sequencing peak plot. (**G**) Comparison map of NCBI sequence of *NRAMP1* gene.

Figure 3A–E show the PCR-RFLP verification of the BB genotype of *NRAMP1* in Shanghai white pigs, Fengjing pigs and Shawutou pigs; three types of banding (AA, BB and AB) were verified in Pudong white pigs, and one type of banding (AB) were verified in Pudong white pigs. It was found that the sequence of the BB-type individuals was consistent with that of Upper AY368473.1 in NCBI. The AA-type and AB-type individuals had a mutation site, C → A, which was consistent with the enzyme digestion results (Figure 3F,G).

**Figure 4:**
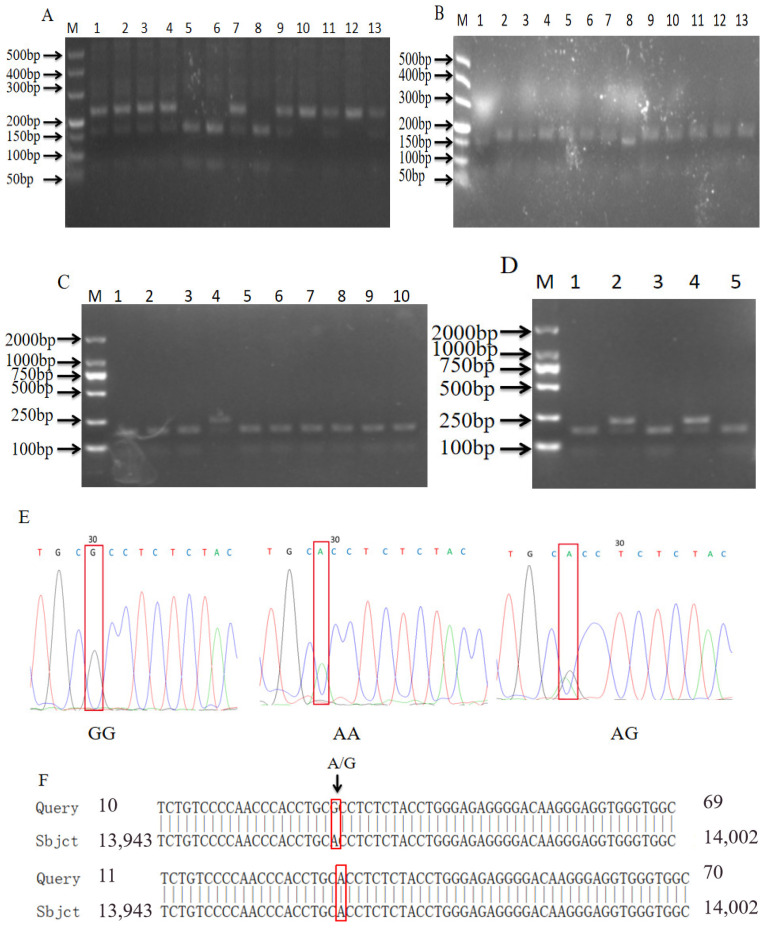
Enzymatic digestion and sequencing results of *MUC4* gene. (**A**–**E**) Enzymatic digestion results of Shawutou pig (**A**), Meishan pig (**B**) and Pudong white pig (**C**,**D**). (**E**) Sequencing peak plot. (**F**) Comparison map of NCBI sequence of *MUC4* gene.

As shown in Figure 4A–D, we found that the *MUC4* genes of the Shanghai white pigs, Fengjing pigs, Shawutou pigs and Pudong white pigs had three genotypes (AA, GG and AG). Meishan pigs had only one stripe type, GG (Figure 4E); the resistance allele (A) could not be digested by *HhaI* as type AA, whereas the susceptibility allele was digested into two fragments as type GG. When two genotypes coexist, the resistant and sensitive alleles are AG-type. The sequence of AA-type individuals was consistent with the sequence of KT966749 in NCBI, which was defined as the wild type. Compared to the GG type, it was found that a single-base mutation of G → A was consistent with the enzyme digestion results (Figure 4F). 

**Figure 5:**
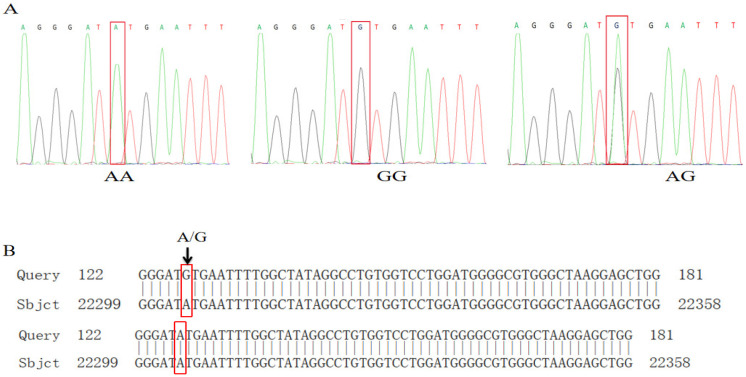
Sequencing results of *MUC13* gene. (**A**) Sequencing peak plot; (**B**) comparison map of NCBI sequence of *MUC13* gene.

Sequencing of the amplified PCR products of the *MUC13* gene was performed on the above pig breeds as shown in Figure 5A. There was a single-base mutation of G → A in position 243 of the *MUC13* gene by comparison of the sequences with JN613418.1 on the NCBI database (Figure 5B). The sequenced results showed that the *MUC13* genes of the Shanghai white pigs had two genotypes (AA and AG), and the genotypes of the mutation in this position were defined as GG types. The peak graphs of four samples showed double peaks at this position in the *MUC13* gene of Shanghai white pigs, defined as AG types. Only one type of banding (GG) was verified in Fengjing pigs, Shawutou pigs and Meishan pigs, and two types of banding (AG and GG) were verified in Pudong white pigs.

### 3.3. Genotype and Allele Frequency Analysis

In Table 1, it is shown that Shanghai white pigs had one allele and two genotypes of *TAP1*. The number and genotype frequency of the AG and GG genotypes were 17 (0.57) and 13 (0.43), respectively; and the frequency of the disease resistance allele G was 0.72, whereas the frequency of the susceptibility allele A was 0.28. The number and genotype frequency of the AA, AG and GG genotypes of *TAP1* in Fengjing pigs were 3 (0.1), 11 (0.37) and 16 (0.53), respectively; the frequencies of the disease resistance allele G and the sensitive allele A were 0.72 and 0.28, respectively. In Shawutou pigs, the number of AA, AG and GG genotypes of *TAP1* was 1 (0.03), 19 (0.63) and 10 (0.33), respectively; the frequency of the disease resistance allele G was 0.65, whereas the frequency of the susceptibility allele A was 0.35. The number of AA, AG and GG genotypes of the *TAP1* gene in Meishan pigs was 11 (0.37), 15 (0.50) and 4 (0.13), respectively; the frequency of the disease resistance allele G was 0.38, whereas the frequency of the susceptibility allele A was 0.62. In the Pudong white pigs, the number of AG and GG genotypes was 13 (0.43) and 17 (0.57), respectively. and the frequencies of the disease resistance allele G and sensitive allele A were 0.78 and 0.22, respectively. In the five breeds, the frequency of detected AA types of *TAP1* was low, and overall, allele G was the dominant allele.

Table 2 shows that only Pudong white pigs were detected with two genotypes, AG and GG, among the samples from all pig breeds tested, while the other pig breeds with the *FUT1* gene were detected only with the sensitive genotype, GG type, with allele G as the dominant allele.

Table 3 shows the detection of *NRAMP1* in the five breeds using the PCR-RFLP method. There was no disease resistance gene type AA was detected in Shanghai white pigs, Shawutou pigs and Fengjing pigs. And the sensitive gene type BB was only detected in the three aforementioned breeds, with allele B as the dominant allele. The numbers of AA, AB and BB genotypes of the *NRAMP1* in Meishan pigs were 2 (0.07), 27 (0.9) and 1 (0.03), respectively. One genotype (AB) was detected in the *NRAMP1* of Pudong white pigs.

Table 4 shows that one pair of alleles and three genotypes of *MUC4* were detected in Shanghai white pigs, Fengjing pigs, Shawutou pigs and Pudong white pigs. The number and frequency of the AA, AG and GG genotypes of *MUC4* in Shanghai white pigs were 1 (0.03), 4 (0.13) and 25 (0.83), respectively. Among them, G was the dominant allele. The number and frequency of the AA, AG and GG genotypes of *MUC4* in Fengjing pigs were 10 (0.33), 19 (0.63) and 1 (0.03), respectively; A was the dominant allele. The number and frequency of the AA, AG and GG genotypes of the *MUC4* gene in Shawutou pigs were 6 (0.2), 14 (0.47) and 10 (0.33), respectively, and G was the dominant allele. The number and frequency of the AA, AG and GG genotypes of the *MUC4* gene in Pudong white pigs were 2 (0.07), 2 (0.07) and 26 (0.86), respectively, and G was the dominant allele. The GG genotype was detected for *MUC4* in Meishan pigs.

Table 5 shows that a total of one pair of alleles and two genotypes of the *MUC13* gene were detected in Shanghai white pigs and Pudong white pigs. The GG genotype was detected in Fengjing pigs, Shawutou pigs and Meishan pigs. The frequency and number of AA and AG genotypes were 26 (0.87) and 4 (0.13) in Shanghai white pigs, in which A was the dominant allele. The frequency and number of AG and GG types were 4 (0.13) and 26 (0.87) in Pudong white pigs, with G as the dominant allele.

### 3.4. Genetic Polymorphism Analysis of TAP1, FUT1, NRAMP1, MUC4 and MUC13 Genes

Table 6 shows that the polymorphism content (PIC) of the *TAP*1 gene of Shanghai white pigs, Fengjing pigs, Shawutou pigs, Meishan pigs and Pudong white pigs was in the range of 0.25–0.5, and all of them exhibited moderate polymorphism. The observed heterozygosity Ho, He and PIC values of Shawutou pigs were higher than those of Shanghai white pigs, Fengjing pigs and Pudong white pigs, indicating that their genetic diversity was higher than that of the other three breeds. The χ20.05 (1) < χ2 < χ20.01 (1) and 0.01 < *p* < 0.05 of Shanghai white pigs and Shawutou pigs were significantly different and did not satisfy the Hardy–Weinberg equilibrium. The χ2 < χ20.05 (1) and *p* > 0.05 of Fengjing pigs, Meishan pigs and Pudong white pigs were not significantly different and satisfied the Hardy–Weinberg equilibrium.

The results of enzyme digestion and sequencing of the *FUT1* gene in Shanghai white pigs, Fengjing pigs, Shawutou pigs, Meishan pigs and Pudong white pigs showed that only the GG genotype was detected in Shanghai white pigs, Fengjing pigs, Shawutou pigs and Meishan pigs, and the *FUT1* gene could not undergo further analysis for genetic polymorphism and Hardy–Weinberg equilibrium. In Pudong white pigs, the values of Ho, He and PIC were 0.167, 0.155 and 0.144, respectively; the PIC was less than 0.25, which indicated low polymorphism; χ2 < χ20.05 (1); *p* > 0.05; and the difference was not significant and satisfied the Hardy–Weinberg equilibrium.

The results of enzyme digestion and sequencing of the *NRAMP1* gene in Shanghai white pigs, Fengjing pigs and Shawutou pigs showed that they only had BB genotypes, and they could not be further analyzed for genetic polymorphism and Hardy–Weinberg equilibrium. The values of HO, He and PIC for Meishan pigs and Pudong white pigs were 0.900, 0.508 and 0.375 and 1.000, 0.508 and 0.375, respectively, and the PIC values were all in the range of 0.25–0.5, which was moderately polymorphic, with χ2 > χ20.01 (1) and *p* < 0.01, which was highly significant and did not satisfy the Hardy–Weinberg equilibrium.

The PIC of the *MUC4* gene of Shanghai white pigs and Pudong white pigs was less than 0.25, indicating low polymorphism, and the PIC of the *MUC4* gene of Fengjing pigs and Shawutou pigs was between 0.25 and 0.5, indicating medium polymorphism. GG genotypes were detected in Meishan pigs, and polymorphisms were not calculated. The He and PIC values of Shawutou pigs were higher than those of Fengjing pigs, Shawutou pigs and Pudong white pigs. This indicates that the genetic polymorphism of Shawutou pigs was higher than that of the other three breeds. Analyses revealed that χ2 < χ20.05 (1) and *p* > 0.05 for Shanghai white pigs and Shawutou pigs; the difference was not significant and satisfied the Hardy–Weinberg equilibrium. For, Fengjing pigs, χ20.05 (1) < χ2 < χ20.01 (1) and 0.01 < *p* < 0.05; the difference was significant and did not satisfy the Hardy–Weinberg equilibrium. For Pudong white pigs, χ2 > χ20.01 (1) and *p* < 0.01; the difference was highly significant and did not satisfy the Hardy–Weinberg equilibrium.

The PIC of the *MUC13* gene in Shanghai white pigs and Pudong white pigs was between 0.25 and 0.5, which was moderately polymorphic. χ2 < χ20.05 (1); the difference was not significant and satisfied the Hardy–Weinberg equilibrium (*p* > 0.05). The *MUC13* gene of Fengjing pigs, Shawutou pigs and Meishan pigs had the GG genotype; hence, the polymorphism of the *MUC13* gene in several pig breeds was not calculated.

### 3.5. Determination of Immune Factors in Shanghai White Pigs, Fengjing Pigs, Shawutou Pigs, Meishan Pigs and Pudong White Pigs

The results of the immunity factor measurements of different pig breeds are shown in Table 7. There was no significant difference in the mean levels of the IFN-γ blood immunity factor among Shanghai white pigs, Fengjing pigs, Shawutou pigs, Meishan pigs and Pudong white pigs *(p* > 0.05). The levels of the IL-2 immunity factor in Meishan pigs were markedly higher than those in Shanghai white pigs, Fengjing pigs, Shawutou pigs and Pudong white pigs (*p* < 0.05), and the lowest level was in Fengjing pigs. There was no apparent difference among pig breeds in the IL-6 index (*p* > 0.05). The IL-10 levels of Meishan pigs and Pudong white pigs were obviously higher than those of Shanghai white pigs and Shawutou pigs (*p* < 0.05), and the lowest level was in Shawutou pigs. The IL-12 level of Pudong white pigs was distinctly higher than that of Shanghai white pigs and Fengjing pigs (*p* < 0.05). The IgG level of Meishan pigs was significantly higher than that of Pudong white pigs and Shawutou pigs. The Pudong white pigs had the lowest IgG level. The level of TNF-α of Meishan pigs was significantly higher than that of Shanghai white pigs, Fengjing pigs, Shawutou pigs and Pudong white pigs (*p* < 0.05).

## 4. Discussion

Piglet diarrhea disease is a kind of intestinal infectious disease mainly in newborn and young piglets, with the promotion of large-scale and intensive production modes. It has become a problem that cannot be ignored in pig production. Factors contributing to diarrhea include pathogenic bacteria, feeding and genetic factors. The first two can be reduced by vaccination and better management to reduce their impact on diarrhea. However, genetic factors can only be modified by selectively breeding for genes associated with resistance to diarrhea. In this study, Pudong white, Meishan, Shawutou, Fengjing and Shanghai white pigs were selected from molecular genetics to screen for polymorphisms in TAP1, FUT1, NRAMP1, MUC4 and MUC13 antidiarrheal genes, to measure some of the immunity indices and to analyze the resistance to disease of each pig breed.

### 4.1. Polymorphism of Antidiarrheal Genes

In our study, exon 3 polymorphisms of the *TAP1* gene in five local pig breeds of Shanghai, China, were analyzed. We found that three genotypes (AA, AG and GG) with G as the dominant allele were detected in both Fengjing pigs and Shawutou pigs. The AA genotype gene was not detected in Shanghai white pigs, which may be due to the fact that it was the result of breeding with a foreign pig breed. In the work of Zhang et al., the AA genotype was detected in Pudong white pigs [6], which was inconsistent with the results of our study. This may be due to the insufficiently large sample size of our study and will be verified at a later stage by increasing the sample size of the Pudong white pigs. Zhao et al. reported that the mutation of G729A in the *TAP1* gene of the Sutai pigs significantly affected the expression of mRNA and that the antidiarrhea resistance of individuals with the GG genotype was significantly higher than that of individuals with the other two genotypes [15]. Therefore, the GG genotype of the *TAP1* gene could be a candidate genotype for resistance to diarrheal infection.

The results of the M307 locus of the *FUT1* gene in this experiment showed that only one non-disease-resistant base GG genotype was present in the local breeds of Shanghai white pigs, Fengjing pigs, Shawutou pigs and Meishan pigs. Bao et al. reported that domestic local breeds contained only GG genotypes, whereas foreign and domestic cross-bred breeds of pigs contained AA, AG and GG genotypes in the M307 locus of the *FUT1* gene, with GG being the absolutely dominant genotype [16]. This aforementioned finding is similar to the result of our study. The analysis speculated that the disease resistance allele A of the *FUT1* gene had an extremely skewed distribution in Chinese local pig breeds, and the AA disease resistance genotype might have originated from foreign pig breeds. This reason may be that these local pig breeds in Shanghai have less genetic exchange with domestic and foreign pig breeds and have a unique genetic resourcefulness. Notably, AG and GG genotypes of the *FUT1* gene were detected in Pudong white pigs. Similar to this study, Zhang et al. reported that the AG and GG genotypes of the FUT1 gene were also detected in Congjiang pigs from Guizhou, China [17]. This may be due to the fact that the Pudong white pigs as a native breed and foreign pig breeds have crossbred. Wang et al. also reported that the mtDNA haplotypes H_34 and H_35 carried by the Pudong white pig, which belongs to the Taihu Lake region pig branch, overlapped considerably with those of the European domestic pig [18]. This is obviously speculation on our part, but the topic should merit further research in the future.

The *NRAMP1* gene, as a relatively conserved gene, is known to affect animal autoimmunity and is resistant to Salmonella and a variety of intracellular parasitic pathogens [19]. In this study, no AA type was detected in three pig breeds (Shanghai white pigs, Fengjing pigs and Shawutou pigs), and only the allele BB type was detected. This may be due to the gradual elimination of individuals with this gene in the three Shanghai pig breeds during long-term evolutionary selection. By contrast, three genotypes (AA, AB and BB) were detected in Meishan pigs, which is consistent with the results of Chen et al. on large white pigs and land pigs [8]. The two genotypes AA and AB were detected in two other pig breeds. This may be because the BB genotype is not dominant or because of its inferiority or the insufficient number of samples used in the study. The reason that only one genotype, AB, was detected in the Pudong white pigs could be that the two genotypes AA and BB were disadvantageous genotypes in Pudong white pigs and as a result were not retained in the selection process.

A previous study has shown that the *MUC4* gene can be a candidate gene for F4ab/F4ac resistance and that G is the allele controlling F4ac coliform resistance [12]. Our polymorphism study of the 243rd site polymorphism of the 17th intron of the *MUC4* gene in five local pig breeds of Shanghai revealed that one allele, with three genotypes (AA, AG and GG), was detected in this site. Three genotypes were detected in Shanghai white pigs, Fengjing pigs, Shawutou pigs and Pudong white pigs, and only the antidiarrheal GG genotype was detected in all samples from Meishan pigs. G was the dominant allele in Shanghai white pigs, Shawutou pigs and Meishan pigs, and A was the dominant allele in Fengjing pigs and Pudong white pigs. There were some differences between the five breeds of pigs at the molecular level, and these differences were analyzed as possibly the cause of differences in selection, where the degree of genetic variability varied due to different indirect selection pressures on the different alleles during the selection process. Liu et al. [20] found that large white pig individuals with the GG genotype of the *MUC4* gene had higher resistance to ETEC F4ab/F4ac infection than those with other genotypes. The presence of GG-type resistance genes in all five pig breeds in this study can be selected to achieve population resistance to ETEC F4ac infection.

The *MUC13* gene is localized between Sw207 and S0075 in region 41 of the long arm of pig chromosome 13 [21]. A relevant study has shown that the *MUC13* gene is one of the genes determining susceptibility or resistance to F4ac diarrhea in piglets [10]. In this study, we investigated the intron 2 polymorphism of the *MUC13* gene in five local pig breeds, namely Shanghai white pigs, Fengjing pigs, Shawutou pigs, Meishan pigs and Pudong white pigs, in the Shanghai area and found that two genotypes (AA and AG) were detected in the Shanghai white pigs. The resistance gene, GG type, was detected in the Fengjing pigs, Shawutou pigs and Meishan pigs in this study. It is possible that individuals with the gene were gradually retained in both pig breeds over a long period of evolutionary selection, creating populations with the disease resistance gene. Both AG and GG genotypes were detected in Pudong white pigs. Ren et al. genotyped an SNP in *MUC13* accurately distinguishing pigs susceptible or resistant (GG) to ETEC F4ac [22]. Ruan et al. also reported that the *MUC13* antidiarrhea G allele was selected for in Duroc pigs and successfully selected for only the antidiarrhea GG type [14]. In the current study, all the pig breeds had the G allele, which can be selected to make all the breeds GG-type for this antidiarrhea gene.

### 4.2. Genetic Polymorphism Analysis of TAP1, FUT1, NRAMP1, MUC4 and MUC13 Genes in Shanghai White Pigs, Fengjing Pigs, Shawutou Pigs, Meishan Pigs and Pudong White Pigs

As the detection parameters of genetic variation within a population, the values of PIC, Ho and He reflect the homogeneity of individuals within a population. The higher the values, the greater the genetic variability, the greater the adaptability to the environment, the greater the selection potential and the better the effect of genetic breeding. Conversely, less heterozygosity and high genetic purity in the population can be regarded as a pure line to be utilized [23]. The PICs of the *TAP1* gene in this study were in the range of 0.25–0.5, and all of them were moderately polymorphic. The Ho and He values of *TAP1* gene of Shawutou pigs were higher than those of Shanghai white pigs, Fengjing pigs and Pudong white pigs. This indicated that Shawutou pigs were rich in genetic diversity and had a stronger ability to adapt to the environment and a high genetic potential. The *TAP1* gene did not satisfy the Hardy–Weinberg equilibrium in Shanghai white pigs and Shawutou pigs and satisfied the Hardy–Weinberg equilibrium in Fengjing pigs, Meishan pigs and Pudong white pigs, which indicated that the *TAP1* gene was basically in a state of free mating in Fengjing pigs. This means that population selection had no effect on the *TAP1* gene and had no effect on the present selection traits. This is consistent with the results of previous studies on the *TAP1* gene in Duroc, large white, long white, Fengjing, Meishan and Pudong white pigs, in which the Hardy–Weinberg equilibrium is satisfied.

For Shanghai white pigs, Fengjing pigs, Shawutou pigs and Meishan pigs, *FUT1* had only GG genotypes, so their genetic polymorphisms were not analyzed in this study. Two genotypes were detected in the *FUT1* gene of Pudong white pigs; the genetic polymorphism was analyzed, resulting in a PIC < 0.25. The lower the PIC, the less heterozygous the population and the higher the genetic purity. This value indicated a low degree of polymorphism, and the *FUT1* gene of the Pudong white pigs satisfied the Hardy–Weinberg equilibrium.

Enzymatic cleavage and sequencing of the *NRAMP1* genes of Shanghai white pigs, Fengjing pigs and Shawutou pigs showed only BB genotypes; therefore, their genetic polymorphisms were not analyzed in this study. The PIC values of Meishan pigs and Pudong white pigs were in the range of 0.25–0.5; both were moderately polymorphic, and neither satisfied the Hardy–Weinberg equilibrium. In this study, we found that the Meishan pigs’ and Pudong white pigs’ *NRAMP1* genes had a high Ho, which suggests that the genetic diversity of the two breeds is more abundant. This may be due to the fact that some properties of heterozygous individuals are higher than those of pure individuals. This also makes the pure individuals gradually eliminated during long-term natural selection and artificial breeding, while the heterozygous individuals are retained more. The PIC indicated moderate polymorphism, which further indicated that intron 6 polymorphism of the *NRAMP1* gene was not abundant in the two pig breeds.

The GG genotype was detected in *MUC4* of Meishan pigs, for which no polymorphism was analyzed. The PIC of the *MUC4* gene of Shanghai white pigs and Pudong white pigs indicated low polymorphism, while the PIC of the *MUC4* gene of Fengjing pigs and Shawutou pigs indicated medium polymorphism. The He values of Shawutou pigs were higher than those of the other three pig breeds, which was consistent with the trend of the PIC values, indicating that Shawutou pigs have higher genetic polymorphisms, which can be used as marker-assisted selection for breeding for disease resistance. The lower PIC of the *MUC4* gene in Shanghai white pigs and Pudong white pigs indicated lower genetic variability, but the analysis found that Shanghai white pigs and Shawutou pigs satisfied the Hardy–Weinberg equilibrium. And Fengjing pigs and Pudong white pigs did not satisfy the Hardy–Weinberg equilibrium. This result suggests that artificial selection has had a significant effect on the distribution of *MUC4* genes in the F4ab/F4ac *E. coli* resistance breeding base population of Fengjing pigs and Pudong white pigs. The PIC of the *MUC13* gene in Shanghai white pigs and Pudong white pigs indicated low polymorphism and satisfied the Hardy–Weinberg equilibrium. The sequencing results of the *MUC13* gene in Fengjing pigs, Shawutou pigs and Meishan pigs showed only the GG genotype. Its genetic polymorphism was not analyzed in this study. Some pig breed loci deviated from the Hardy–Weinberg equilibrium in this experiment, and deviation from this equilibrium may be produced due to insufficient sample size in population studies or excessive inbreeding accumulation within a population [24]. In this study, the sample sizes of five local pig breeds of Shanghai lines reached statistical significance, yet the results of analyzing the genetic polymorphisms and heterozygosity of the five breeds showed that there was excessive inbreeding accumulation and partial allelic deletion in the population. Further analysis of the chain imbalance in the populations was mainly due to the high inbreeding or genetic drift in the current populations of five breeds during the preservation process, which was related to the later breed improvement of the above pig breeds.

### 4.3. Immune Factors of Shanghai White Pigs, Fengjing Pigs, Shawutou Pigs, Meishan Pigs and Pudong White Pigs

IgG is the main immunoglobulin class produced during the immune response against foreign antigens. The status of the humoral immunity of an organism can be represented by determining the IgG level in serum [25]. IL-2, IL-6, IL-10 and IL-12 have important roles in the immune system of the organism, and IL-2 mainly activates the immune system [26]. IL-6 and IL-10 are mainly involved in the inflammatory response [27,28], and IL-12 mainly promotes the proliferation of T cells and NK cells [29]. As a cytokine with strong antiviral, antitumor and immunomodulatory effects, IFN-γ also plays an important role in an organism’s immune system [30]. TNF-α belongs to the superfamily of tumor necrosis factors; it not only can inhibit viral replication and pathogen spread and prevent tumorigenesis in the organism but also is an important antimicrobial factor [31]. All of the above serological immune factors are indispensable for organismal immunity, and there is a close link between them as they influence and regulate each other. We analyzed the immune indices of the above five pig breeds. The level of IL-2 in Meishan pigs was significantly higher than that of the other four breeds, and IL-2 has the function of activating T cells. This results in the promotion of the proliferation of B cells and the delivery of antibodies, which is conveniently of significant importance in antiviral responses [26]. It suggests that Meishan pigs may have stronger antiviral effects. There was no difference in IL-6 among pig breeds, but the results of the present study showed that IL-6 index values in Shawutou pigs were lower than those in other pig breeds. Qin et al. reported that serum IL-6 levels of children with non-infectious diarrhea were significantly higher than those of healthy children [32]. This suggests that these levels may be related to the development of non-infectious diarrhea. And low levels of IL-6 may be more dominant in resistance to disease, suggesting that the Shawutou pigs are possibly more resistant to disease.

In this study, the IL-10 levels of Meishan pigs and Pudong white pigs were significantly higher than those of Shanghai white pigs and Shawutou pigs. Some studies have shown that higher IL-10 levels may be more resistant to disease in the animal body [33]. This indicated that Meishan pigs and Pudong white pigs had stronger disease resistance. Higher levels of IL-12, IgG and TNF-α in Meishan pigs indicated that Meishan pigs might be more resistant to diseases than Shanghai white pigs, Fengjing pigs, Shawutou pigs and Pudong white pigs.

Finally, we acknowledge some limitations of this study: the sample size was limited, resulting in a small effect size of the results. Thus, further larger-scale studies with more comprehensive data are needed to obtain more definitive data.

## 5. Conclusions

In this study, the relationships between some antidiarrheal genes and some immune factors in Shanghai white pigs, Fengjing pigs, Shawutou pigs, Meishan pigs and Pudong white pigs were summarized. The analyses provided a preliminary understanding of antidiarrhea in each pig breed, which can provide a certain theoretical basis for further experiments.

## Figures and Tables

**Table 1:** Genotypes and allele frequencies of *TAP1* gene in Shanghai white pigs, Fengjing pigs and Shawutou pigs.

Pig Breeds	Genotype Frequency			Allele Frequency	
	AA	AG	GG	A	G
Shanghai white pigs	0 (0)	0.57 (17)	0.43 (13)	0.28	0.72
Fengjing pigs	0.1 (3)	0.37 (11)	0.53 (16)	0.28	0.72
Shawutou pigs	0.03 (1)	0.63 (19)	0.33 (10)	0.35	0.65
Meisha pigs	0.37 (11)	0.50 (15)	0.13 (4)	0.62	0.38
Pudong white pigs	0 (0)	0.43 (13)	0.57 (17)	0.22	0.78

**Table 2:** Genotypes and allele frequencies of *FUT1* gene in Shanghai white pigs, Fengjing pigs and Shawutou pigs.

Pig Breeds	Genotype Frequency			Allele Frequency	
	AA	AG	GG	A	G
Shanghai white pigs	0 (0)	0 (0)	1 (30)	0	1
Fengjing pigs	0 (0)	0 (0)	1 (30)	0	1
Shawutou pigs	0 (0)	0 (0)	1 (30)	0	1
Meisha pigs	0 (0)	0 (0)	1 (30)	0	1
Pudong white pigs	0 (0)	0.17 (5)	0.83 (25)	0.08	0.92

**Table 3:** Genotypes and allele frequencies of *NRAMP1* gene in Shanghai white pigs, Fengjing pigs and Shawutou pigs.

Pig Breeds	Genotype Frequencies			Allele Frequency	
	AA	AB	BB	A	B
Shanghai white pigs	0 (0)	0 (0)	1 (30)	0	1
Fengjing pigs	0 (0)	0 (0)	1 (30)	0	1
Shawutou pigs	0 (0)	0 (0)	1 (30)	0	1
Meisha pigs	0.07 (2)	0.9(27)	0.03 (1)	0.52	0.48
Pudong white pigs	0 (0)	1 (30)	0 (0)	0.5	0.5

**Table 4:** Genotypes and allele frequencies of *MUC4* gene in Shanghai white pigs, Fengjing pigs and Shawutou pigs.

Pig Breeds	Genotype Frequencies			Allele Frequency	
	AA	AG	GG	A	G
Shanghai white pigs	0.03 (1)	0.13 (4)	0.83 (25)	0.10	0.90
Fengjing pigs	0.33 (10)	0.63 (19)	0.03 (1)	0.65	0.35
Shawutou pigs	0.2 (6)	0.47 (14)	0.33 (10)	0.43	0.57
Meisha pigs	0 (0)	0 (0)	1 (30)	0	1
Pudong white pigs	0.07 (2)	0.07 (2)	0.86 (26)	0.9	0.1

**Table 5:** Genotypes and allele frequencies of *MUC13* gene in Shanghai white pigs, Fengjing pigs and Shawutou pigs.

Pig Breeds	Genotype Frequencies			Allele Frequency	
	AA	AG	GG	A	G
Shanghai white pigs	0.87 (26)	0.13 (4)	0 (0)	0.93	0.07
Fengjing pigs	0 (0)	0 (0)	1 (30)	0	1
Shawutou pigs	0 (0)	0 (0)	1 (30)	0	1
Meisha pigs	0 (0)	0 (0)	1 (30)	0	1
Pudong white pigs	0 (0)	0.13 (4)	0.87 (26)	0.07	0.93

**Table 6:** Genetic polymorphism analysis of *TAP1*, *FUT1*, *NRAMP1*, *MUC4* and *MUC13* genes in Shanghai white pigs, Fengjing pigs and Shawutou pigs.

Genes	Pig Breeds	Genetic Polymorphism Analysis			
		Ho	He	PIC	χ2
*TAP1*	Shanghai white pigs	0.567	0.413	0.324	4.76
	Fengjing pigs	0.367	0.413	0.324	0.29
	Shawutou pigs	0.633	0.463	0.351	4.61
	Meisha pigs	0.500	0.481	0.361	0.10
	Pudong white pigs	0.433	0.345	0.282	2.290
*FUT1*	Shanghai white pigs	/	/	/	/
	Fengjing pigs	/	/	/	/
	Shawutou pigs	/	/	/	/
	Meisha pigs	/	/	/	/
	Pudong white pigs	0.167	0.155	0.141	0.275
*NRAMP1*	Shanghai white pigs	/	/	/	/
	Fengjing pigs	/	/	/	/
	Shawutou pigs	/	/	/	/
	Meisha pigs	0.90	0.508	0.375	19.32
	Pudong white pigs	1.0	0.508	0.375	30
*MUC4*	Shanghai white pigs	0.133	0.183	0.164	2.01
	Fengjing pigs	0.633	0.463	0.351	4.62
	Shawutou pigs	0.467	0.499	0.371	0.08
	Meisha pigs	/	/	/	/
	Pudong white pigs	0.067	0.183	0.164	11.89
*MUC13*	Shanghai white pigs	0.133	0.127	0.117	0.15
	Fengjing pigs	/	/	/	/
	Shawutou pigs	/	/	/	/
	Meisha pigs	/	/	/	/
	Pudong white pigs	0.133	0.127	0.117	0.15

Note: χ^2^ 0.05 (1) = 3.84, χ^2^ 0.01 (1) = 6.63. PIC ≥ 0.5, highly polymorphic; 0.25 ≤ PlC < 0.5, moderately polymorphic; and PIC < 0.25, low polymorphism.

**Table 7:** Analysis of differences in blood immune factors among Shanghai white pigs, Fengjing pigs and Shawutou pigs (unit: pg/mL).

Immune Factor	Pig Breeds				
	Shanghai White Pigs	Fengjing Pigs	Shawutou Pigs	Meishan Pigs	Pudong White Pigs
IFN-γ	24.80 ± 1.33 ^a^	25.97 ± 1.40 ^a^	25.91 ± 1.41 ^a^	24.98 ± 1.37 ^a^	26.66 ± 1.39 ^a^
IL-2	239.59 ± 10.63 ^bc^	207.69 ± 10.55 ^c^	212.75 ± 10.83 ^c^	331.48 ± 11.55 ^a^	254.98 ± 9.10 ^b^
IL-6	627.26 ± 33.32 ^a^	626.74 ± 28.40 ^a^	610.44 ± 31.98 ^a^	650.13 ± 32.77 ^a^	676.45 ± 27.17 ^a^
IL-10	345.10 ± 22.61 ^b^	361.09 ± 21.17 ^ab^	335.41 ± 21.39 ^b^	400.94 ± 13.33 ^a^	389.81 ± 14.58 ^a^
IL-12	159.89 ± 8.45 ^b^	160.97 ± 7.38 ^b^	169.22 ± 9.15 ^ab^	180.18 ± 8.19 ^ab^	190.53 ± 8.97 ^a^
IgG	475.08 ± 16.97 ^ab^	482.82 ± 17.53 ^ab^	439.75 ± 16.18 ^b^	487.27 ± 17.98 ^a^	283.02 ± 17.23 ^c^
TNF-α	142.64 ± 6.98 ^b^	145.36 ± 7.20 ^b^	145.92 ± 7.49 ^b^	223.07 ± 6.66 ^a^	146.11 ± 7.12 ^b^

Note: There was a significant difference (superscript letters) in the average number of peers with different shoulder markers (*p* < 0.05).

## Data Availability

Data are contained within the article and Supplementary Materials.

## Supplementary Materials

The following supporting information can be downloaded at: https://www.mdpi.com/article/10.3390/biom14050595/s1, Figure S1: Electrophoresis of PCR products of *TAP*1, *FUT1*, *NRAMP1*, *MUC4* and *MUC13* in Shanghai white pigs, Fengjing pigs, Shawutou pigs, Meishan pigs and Pudong white pigs; Table S1: The body weights and ages of pigs; Table S2: Primer sequences used for PCR in this study.
