# Supplementary material for: Detection and Analysis of Antidiarrheal Genes and Immune Factors in Various Shanghai Pig Breeds
Source: Biomolecules. 2024 May 17;14(5):595. doi: 10.3390/biom14050595 (PMC11117698; doi:10.3390/biom14050595)
Supplement: Supplementary file 1 [file biomolecules-14-00595-s001.zip › Supplementary Figure S1.pdf]

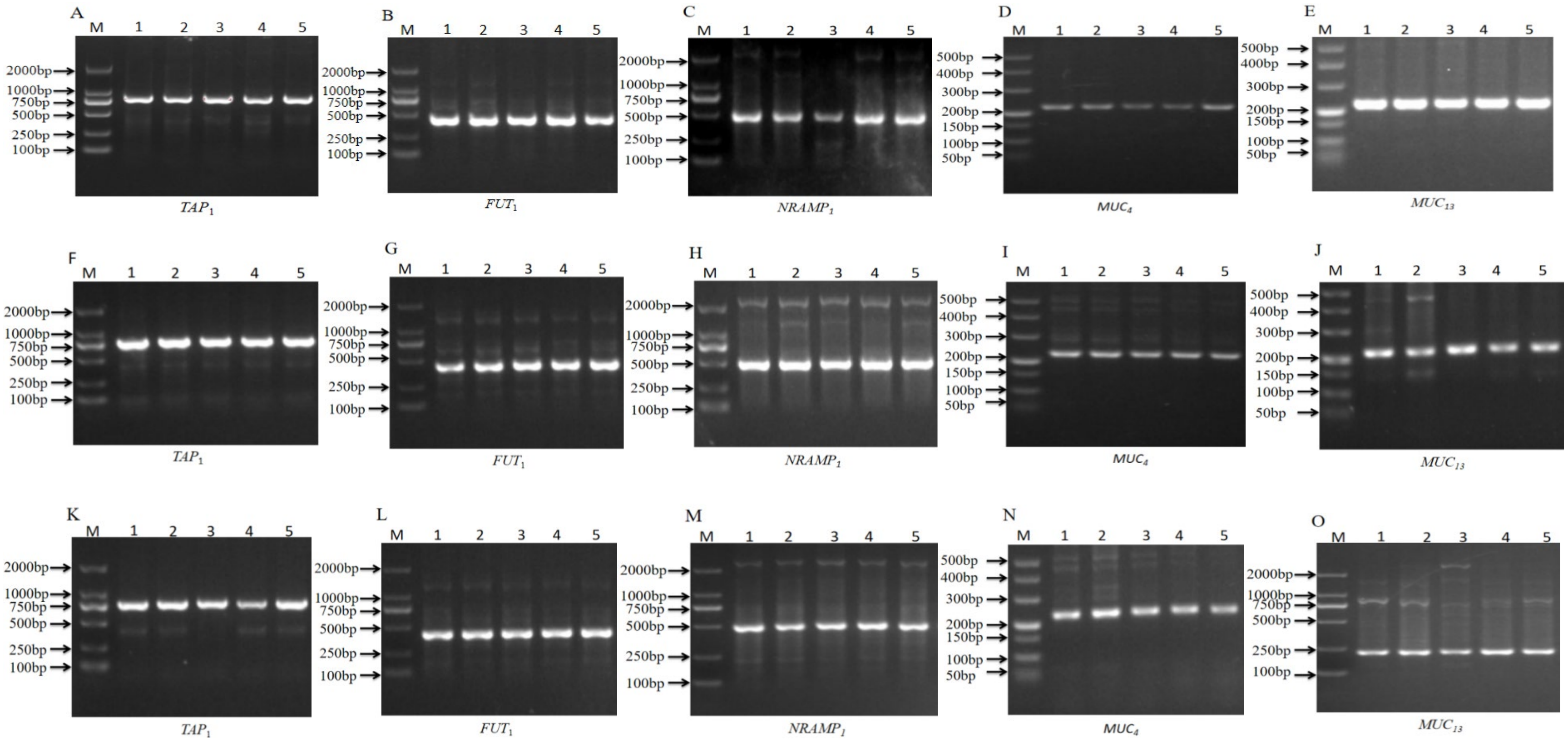
A
M 1 2 3 4 5
2000bp 1000bp 750bp 500bp 250bp 100bp
TAP1
B
M 1 2 3 4 5
2000bp 1000bp 750bp 500bp 250bp 100bp
FUT1
C
M 1 2 3 4 5
2000bp 1000bp 750bp 500bp 250bp 100bp
NRAMP1
D
M 1 2 3 4 5
500bp 400bp 300bp 200bp 150bp 100bp 50bp
MUC4
E
M 1 2 3 4 5
500bp 400bp 300bp 200bp 150bp 100bp 50bp
MUC13
F
M 1 2 3 4 5
2000bp 1000bp 750bp 500bp 250bp 100bp
TAP1
G
M 1 2 3 4 5
2000bp 1000bp 750bp 500bp 250bp 100bp
FUT1
H
M 1 2 3 4 5
2000bp 1000bp 750bp 500bp 250bp 100bp
NRAMP1
I
M 1 2 3 4 5
500bp 400bp 300bp 200bp 150bp 100bp 50bp
MUC4
J
M 1 2 3 4 5
500bp 400bp 300bp 200bp 150bp 100bp 50bp
MUC13
K
M 1 2 3 4 5
2000bp 1000bp 750bp 500bp 250bp 100bp
TAP1
L
M 1 2 3 4 5
2000bp 1000bp 750bp 500bp 250bp 100bp
FUT1
M
M 1 2 3 4 5
2000bp 1000bp 750bp 500bp 250bp 100bp
NRAMP1
N
M 1 2 3 4 5
500bp 400bp 300bp 200bp 150bp 100bp 50bp
MUC4
O
M 1 2 3 4 5
2000bp 1000bp 750bp 500bp 250bp 100bp
MUC13

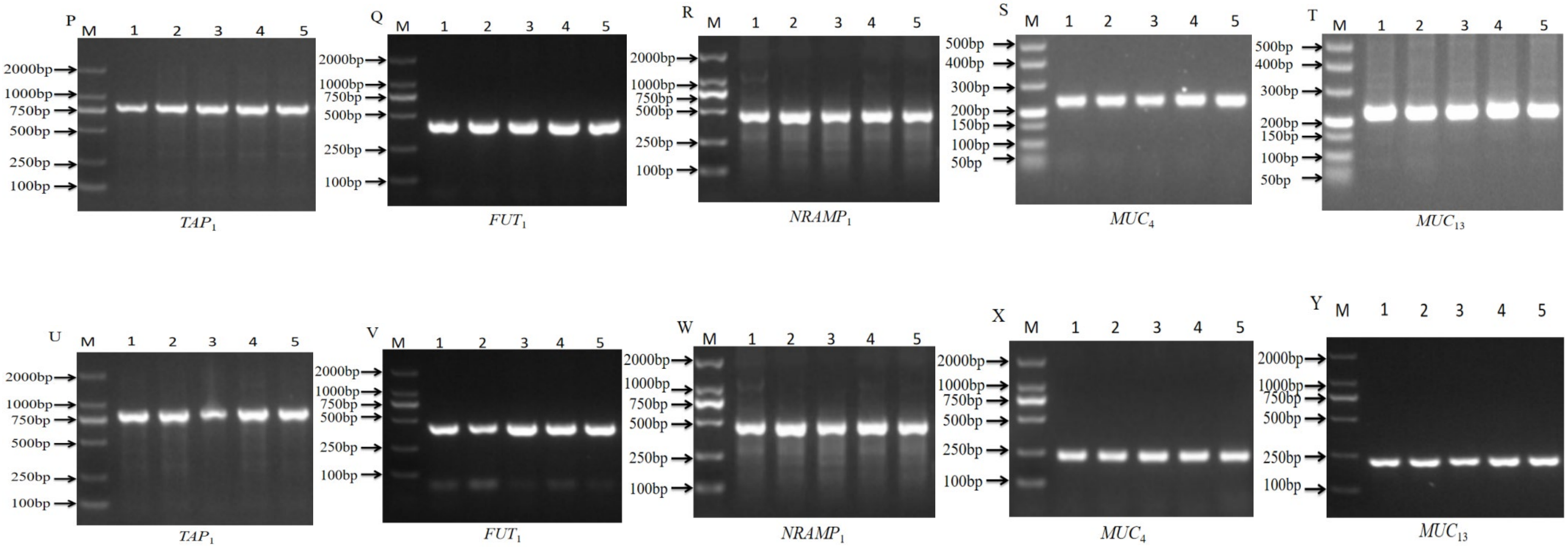

Figure S1. Electrophoresis of PCR products of *TAP₁*, *FUT₁*, *NRAMP₁*, *MUC₄*, and *MUC₁₃* in Shanghai White Pig, Fengjing Pig, Sha Wutou Pig, Meishan pig and Pudong white pig

A-E is the electrophoretic map of 5 genes in Shanghai White Pig, F-J is the electrophoretic map of 5 genes in Fengjing Pig, K-O is the electrophoretic map of 5 genes in Sha Wutou Pig, P-T is the electrophoretic map of 5 genes in Meishan pig, U-Y is the electrophoretic map of 5 genes in pudong white pig. M is DNAMarker, and 1-5 digits are PCR amplification results.
