# Supplementary material for: Detection and Analysis of Antidiarrheal Genes and Immune Factors in Various Shanghai Pig Breeds
Source: Biomolecules. 2024 May 17;14(5):595. doi: 10.3390/biom14050595 (PMC11117698; doi:10.3390/biom14050595)
Supplement: Supplementary file 1 [file biomolecules-14-00595-s001.zip › Supplementary Table S1.pdf]

Table S1. The body weights and ages of pigs.

Pig breeds	Ages (Days)	Weights (Kg)
Shanghai white pigs	238.17 ± 16.31	74.93 ± 5.58
Fengjing pigs	240.23 ± 16.80	77.43 ± 4.19
Shawutou pigs	244.70 ± 16.78	78.57 ± 5.38
Meisha pigs	240.47 ± 18.39	77.50 ± 6.10
Pudong white pigs	237.03 ± 18.06	77.77 ± 4.07
