# Supplementary material for: Detection and Analysis of Antidiarrheal Genes and Immune Factors in Various Shanghai Pig Breeds
Source: Biomolecules. 2024 May 17;14(5):595. doi: 10.3390/biom14050595 (PMC11117698; doi:10.3390/biom14050595)
Supplement: Supplementary file 1 [file biomolecules-14-00595-s001.zip › Supplementary Table S2.pdf]

Table S2. Primer sequences used for PCR in this study.

Gene name	Primer sequences (5-3)	Tm (°C)	Length (bp)
TAP$_{1}$	F: GAAATGTGGATAAGAGCA<br>R: AAACAGACGGATAATGAAAGAGG	767	46
FUT$_{1}$	F: CTTBBTGAACGTCATCAAGABB<br>R: CTTCAGBBAGGGCTBBTTTAAG	421	55
NRAMP$_{1}$	F: GGBBAGCTTBBACAGTCTBBAG<br>R: CGGGGGTACAAAGGGGAAGAAG	483	64
MUC$_{4}$	F: TCTAAAGATGCTGGTGCTAC<br>R: CTGGCTGTATTTCTGTTGTG	220	50
MUC$_{13}$	F: GGAGAGABBAAABBCACAGA<br>R: CTBBTCABBAGCTBBTTAGC	230	60
